# A technology-enhanced model of care for transitional palliative care versus attention control for adult family caregivers in rural or medically underserved areas: study protocol for a randomized controlled trial

**DOI:** 10.1186/s13063-020-04806-0

**Published:** 2020-10-28

**Authors:** Diane E. Holland, Catherine E. Vanderboom, Jay Mandrekar, Bijan J. Borah, Ann Marie Dose, Cory J. Ingram, Joan M. Griffin

**Affiliations:** 1 Department of Health Sciences Research, Mayo Clinic, 200 First St SW, Rochester, MN 55905 USA; 2 The Robert D. and Patricia E. Kern Center for the Science of Health Care Delivery, Mayo Clinic, Rochester, MN USA; 3 Center for Palliative Medicine, Mayo Clinic, Rochester, MN USA

**Keywords:** Caregiver, Care transitions, Hospital discharge, Research

## Abstract

**Background:**

Transitioning care from hospital to home is associated with risks of adverse events and poor continuity of care. These transitions are even more challenging when new approaches to care, such as palliative care, are introduced before discharge. Family caregivers (FCGs) are expected to navigate these transitions while also managing care. In addition to extensive caregiving responsibilities, FCGs often have their own health needs that can inhibit their ability to provide care. Those living in rural areas have even fewer resources to meet their self-care and caregiving needs. The purpose of this study is to test the efficacy and cost-effectiveness of an intervention to improve FCGs’ health and well-being. The intervention uses video visits to teach, guide, and counsel FCGs in rural areas during hospital-to-home transitions. The intervention is based on evidence of transitional and palliative care principles, which are individualized to improve continuity of care, provide caregiver support, enhance knowledge and skills, and attend to caregivers’ health needs. It aims to test whether usual care practices are similar to this technology-enhanced intervention in (1) caregiving skills (e.g., caregiving preparedness, communication with clinicians, and satisfaction with care), (2) FCG health outcomes (e.g., quality of life, burden, coping skills, depression), and (3) cost. We describe the rationale for targeting rural caregivers, the methods for the study and intervention, and the analysis plan to test the intervention’s effect.

**Methods:**

The study uses a randomized controlled trial design, with FCGs assigned to the control condition or the caregiver intervention by computer-generated lists. The intervention period continues for 8 weeks after care recipients are discharged from the hospital. Data are collected at baseline, 2 weeks, 8 weeks, and 6 months. Time and monetary costs from a societal perspective are captured monthly.

**Discussion:**

This study addresses 2 independent yet interrelated health care foci—transitional care and palliative care—by testing an intervention to extend palliative care practice and improve transition management for caregivers of seriously ill patients in rural areas. The comprehensive cost assessment will quantify the commitment and financial burden of FCGs.

**Trial registration:**

ClinicalTrials.gov NCT03339271. Registered on 13 November 2017.

Protocol version: 11.

**Supplementary information:**

**Supplementary information** accompanies this paper at 10.1186/s13063-020-04806-0.

## Introduction

### Scientific background and explanation of rationale

The literature increasingly recognizes the profound physical, emotional, social, and financial impact of caring for a loved one with a life-limiting illness in the home environment. When palliative care is introduced during a hospital stay, family caregivers (FCGs) often are expected to take on palliative care responsibilities after discharge, which include managing the new model of palliative care while ensuring the safety and quality of care for the recipient. The recognition that care transitions are fraught with the risk of adverse events (AEs) has led to a call for increased support for the FCG transition experience [1].

An effective approach to improving quality of care and mitigating the risk associated with transitions is the use of advanced practice nurses (APNs) to provide continuing care across settings. The APN interacts directly with patients and families, both in the hospital and in the home, to promote optimal care transitions from acute to community-based care and ensures that continuing care needs are met [2–7]. Providing transitional care services at home is not always viable, however, especially for patients and family members living in rural areas. Although palliative care services are rapidly expanding, specialized practice is still concentrated in urban medical centers. For APNs to travel from an urban medical center to patients’ rural homes is time consuming and cost prohibitive, which makes the provision and evaluation of transitioning palliative care services to rural settings problematic.

With a disproportionate and increasing population of older adults in rural communities, unmet transitional care needs for patients and caregivers are expected to expand. For example, in Minnesota, 30% of the state’s residents live in rural communities, yet 41% of rural residents are older than 65 years, which has important implications for the effective delivery of palliative and transitional care [8]. Numerous health-related disparities exist between rural and urban adults, which is demonstrated by a high incidence of illness and poor health among rural adults. Rural adults are more likely to be without regular primary and specialty health care and to have limited access to emergency services. Rural residents are more likely to have chronic illness or disability and to report being in poor health. Despite more health problems, rural elders tend to use fewer health services because of limited availability of services and travel requirements [9].

The use of health information technology (HIT) and communication systems has been described as the single most important way to equalize the differences in resource availability between rural and urban areas [10]. HIT may help address unmet needs for rural caregivers delivering palliative care during transitions from hospital to home, decrease their risk of adverse health outcomes, and may reduce the direct and indirect costs of care incurred by patients and families. The emerging recognition of the importance of and challenges to rural FCGs in successful care transitions and palliative care demands specific attention to caregivers’ risks of impaired health and poor quality of life to minimize burden and risk of depression [11, 12]. Our proposed study addresses a considerable gap in research on FCGs during transitions of care from hospital to home, as well as a knowledge gap in supporting the 40 million caregivers in the USA [13].

### Objectives

This study uses a novel video intervention to support FCGs living in rural areas through the transition of their loved ones from hospital to home. It is burdensome in terms of time and cost for patients to travel to urban areas for treatment and for nurses to travel from an urban medical center to patients’ rural homes. Thus, the provision and evaluation of transitioning health services to palliative care in rural settings is of critical importance [14, 15]. Our intervention is customized to address the unique needs of rural FCGs, rather than FCG needs as an adjunct to patients’ needs. Our proposed care model draws from existing strategies shown to positively influence FCG outcomes in home-based palliative care practices [16–20] and transitional care models [21–23]. Previous research has shown that enhancing FCGs’ knowledge and skills while attending to their own health needs allows them to continue to provide the best care to their loved ones in the home setting while maintaining their own health and wellness. This evidence has informed the basis of our intervention [24–26]. Few, if any, studies have used video visits in a transitional care model to address the needs of rural FCGs of palliative care patients as they transition from urban medical centers to their homes in rural, underserved areas; the caregiving cost from a societal perspective also has not been evaluated.

Unlike a manualized intervention in which the components may not match the FCG’s specific needs, the transitional palliative care intervention is based on evidence-based transitional and palliative care principles but is customized for each FCG and their challenges in both providing continuity of care in rural areas and maintaining their own health. The intervention provides ongoing FCG teaching, guidance, and counseling, and enhances FCGs’ caregiving knowledge and skills while attending to their own health needs. The specific aims of this study are to test the efficacy of this new care model by comparing the control condition with an HIT-enhanced transitional palliative care intervention for FCGs. The outcomes (aims) measured are (1) caregiving preparedness, communication with clinicians, and satisfaction with care for rural FCGs as the care recipient moves from hospital care to home; (2) FCG quality of life, burden, coping skills, and depression; and (3) health care costs.

We hypothesize that, compared with the control conditions, (1) rural FCGs assigned to the intervention will report increased preparedness for caregiving, improved communication with clinicians, and greater satisfaction with care during the study enrollment period; (2) FCGs in the intervention group will report increased quality of life and coping and decreased depression and burden during the study enrollment period; and (3) there is a net decrease in direct and indirect costs for 6 months after the addition of transitional palliative care for rural FCGs in the intervention group.

## Methods

The protocol described was registered with ClinicalTrials.gov on November 13, 2017 (No. NCT03339271) (Supplemental Table 1). Primary findings from this study will be reported to ClinicalTrials.gov. The Mayo Clinic Institutional Review Board (IRB) approved the study on October 30, 2017 (No. 17-005188). Day-to-day support is provided by the principal investigator (PI). The study coordinator (SC) is responsible for identifying potential recruits and obtaining consent. Our study team has weekly meetings to regularly review protocol fidelity and to discuss and record study progress. Investigators meet monthly to discuss trial progress. The PI works with the team to develop and submit annual progress reports summarizing study progress to the study funders. All unexpected or serious AEs are reported by the PI to the IRB. The PI and study team adhere to IRB policies and procedures with regard to any unanticipated issues involving risk to participants and are required to report any protocol violations.

A Data Safety Monitoring Committee (DSMC) was established to closely monitor the trial for participant safety. The DSMC meets biannually and more frequently if an issue arises. AEs are also reported to the DSMC.

### Trial design and allocation

The study design is a randomized controlled trial, and FCGs are assigned to either the intervention group or the control condition group by a computer-generated randomization list accessible only to the SCs. The Palliative Care hospital consulting service is not notified of study participation; therefore, they are blinded to both participation and group allocation. Data analysts are blinded to group allocation for analysis purposes. Investigators do not anticipate any requirement for unblinding because the nurse interventionists are not blinded.

### Setting

Participants are recruited from a large, comprehensive academic medical center in the upper Midwest by the SCs. The medical center has approximately 62,400 inpatient admissions annually. The majority of patients (80%) come from a 120-mile radius of the study site. Of the 31 counties in the hospital’s major catchment area, 26 are designated as *rural* or federally designated *medically underserved areas* [27, 28]. A Palliative Care consulting service is available to any patient hospitalized at 1 of the 2 hospitals associated with the medical center. The Palliative Care service is organized into 5 teams. The interdisciplinary teams are composed of a physician, an APN, a nurse, a social worker, a chaplain, a music therapist, and a pharmacist. An average of 6 to 10 new palliative care consults occur per day across all 5 teams.

### Eligibility

Our target population includes community-dwelling adult FCGs living in rural or medically underserved areas in Minnesota, Wisconsin, and Iowa and who are providing care to a loved one with a serious life-limiting illness and receiving palliative care while in the hospital (Table 1).

**Table 1:**
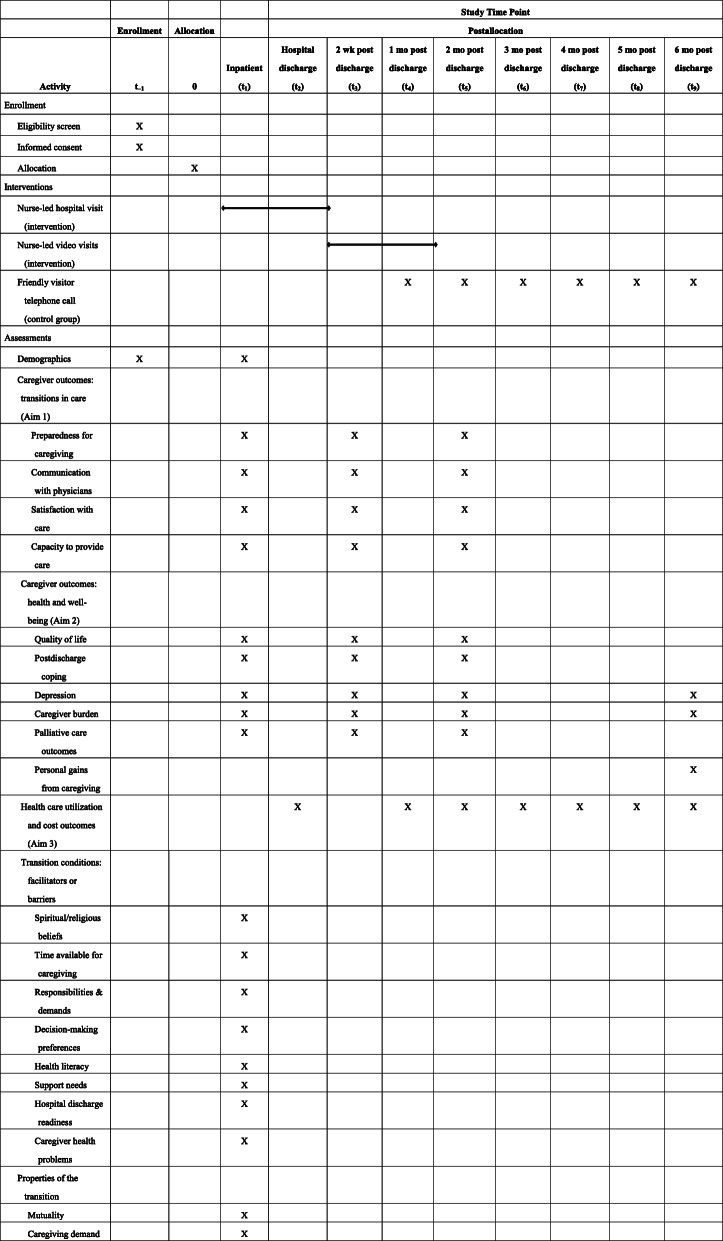
Technology-enhanced transitional palliative care for family caregivers: schedule of randomized controlled trial activities (enrollment, study interventions, and participant assessments)

#### FCGs

An FCG is broadly defined as the person who self-identifies as the family member or unpaid friend who is the primary informal caregiver for a patient with a life-limiting illness. The FCG may or may not be a member of the care recipient’s nuclear family. Any adult FCG (≥ 21 years) of an adult patient hospitalized at 1 of the 2 medical center hospitals who receives an in-hospital palliative care consult and who lives in a Minnesota, Wisconsin, or Iowa county that is designated as medically underserved or rural is invited to participate.

FCGs are remunerated commensurate with the time requirements for data collection and time spent in video visits with the palliative care APN and SC. FCGs in the intervention group are remunerated for data collection activities and for intervention activities. FCGs in the control group are remunerated for data collection activities.

#### Care recipients

Any adult patient (≥ 21 years) hospitalized at 1 of the 2 medical center hospitals who receives an in-hospital palliative care consult and lives in a community in Minnesota, Wisconsin, or Iowa that is designated as medically underserved or rural is invited to participate. Patients with left ventricular assist devices, documented chronic pain, use of home infusion pain pumps, or documented addictive behaviors are excluded from this study because of the unique caregiving needs not addressed by this study. Although our target population is community-dwelling adult FCGs providing care to a loved one who has a serious life-limiting illness and is new to palliative care, the care recipient also must consent or assent because their health and care are discussed during the nurse/FCG video visits. There are no intervention activities for care recipients. Informed consent or assent of the care recipient is obtained by a trained SC after a conversation outlining the risks, benefits, and goals of the investigation.

### Intervention

After a palliative care consult, the SC contacts the FCG to inform them of the study and then meets with them to discuss study eligibility. The SC uses an IRB-approved informational brochure to introduce the study and an IRB-approved consent form. Interpreters are available for persons who have difficulty with the English language.

The SC visits the FCGs and care recipients before hospital discharge to further explain the study, obtain consent, and collect baseline data. If a care recipient lacks the cognitive capacity to provide informed consent, the care recipient’s documented legal authorized representative signs the consent form. As necessary, assent of the care recipient is documented on a separate assent form. After consent, the SC reveals the randomized group assignment.

#### Experimental group: technology-enhanced transitional palliative care intervention

Consistent with other transitional care models [29, 30], the intervention begins while the care recipient is in the hospital and continues for 8 weeks after discharge. The intervention includes in-hospital and in-home components. The in-hospital component includes visits by the nurse interventionists with the caregiver. The in-home component is conducted using video visits and supplemented by telephone calls and texts, if necessary, as described below.

The intervention incorporates key objectives of both transitional and palliative care. Over the course of the 8-week intervention, the interventionist—an experienced, certified palliative care registered nurse—develops an individualized and modifiable FCG plan of care based on their assessment of FCG risks, needs, strengths, and preferences (Table 2) [24, 26, 31–35]. The unique plan of care to support FCGs includes the following: (1) identifying personal strengths they can use in providing care, (2) caregiving education and support for the FCG to meet the care recipient’s needs during the initial post-discharge period, and (3) a holistic wellness plan to meet the FCG’s own needs, including self-care (preventive and health-related physical needs) and emotional, spiritual, and social needs during the transition period. Once the direct caregiving needs stabilize, the nurse assists the FCG in identifying and addressing their self-care needs. The nurse assists the FCG in implementing the plan of care and works with the FCG to refine the plan of care to accommodate changing caregiving and self-care needs throughout the 8-week intervention. Nurse interventionists provide FCG advocacy, teaching, guidance, and counseling using in-person visits (during the hospital stay), and video visits and telephone contacts at home.

**Table 2:**
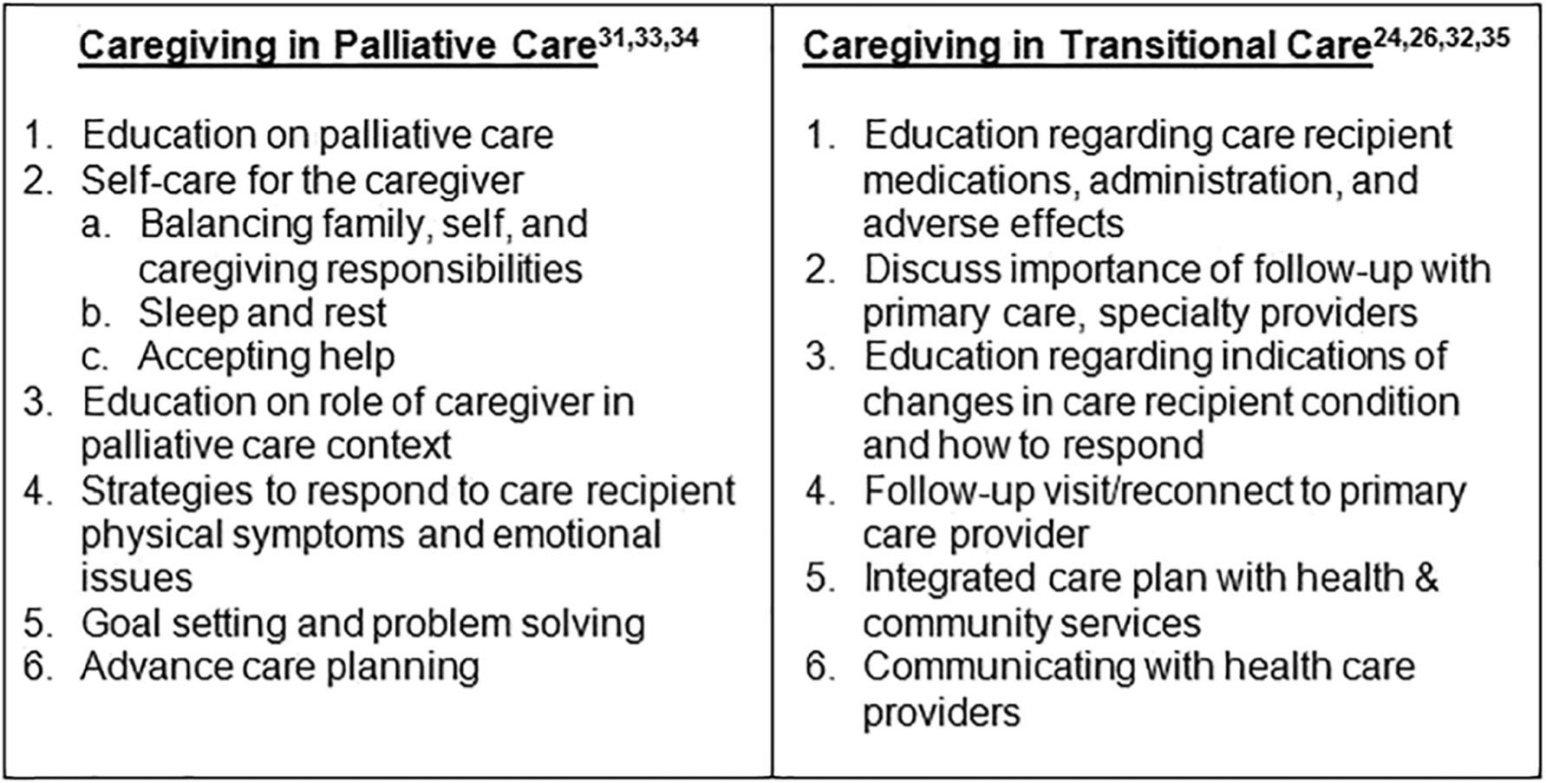
Technology-enhanced transitional palliative care for family caregivers: intervention objectives

The nurse interventionists, with guidance from study investigators and other study palliative care team members (physician, pharmacist, and social worker), review each case at least weekly and make recommendations for the interventionist. Study team meetings focus collectively on identifying and meeting the unique caregiving and self-care needs of each FCG, incorporating tenets of both transitional care and palliative care (Table 2).

##### Part 1: In-hospital intervention

The study nurse makes the first in-hospital visit with the FCG within 24 h of study enrollment, along with twice-weekly visits with the FCG during the remainder of the care recipient’s hospital stay. The study nurse has access to all baseline data collected to assist with assessment and care planning (Table 3). During the in-hospital visits, the nurse (1) assesses the FCG’s knowledge and skills for caregiving; (2) begins transitional care planning by working with the FCG, the palliative care consulting service and acute care staff, and local community service providers in the rural community or surrounding area; and (3) assesses the FCG’s physical and emotional self-care needs, including the involvement of secondary caregivers and/or formal service providers to support the FCG.

**Table 3:** Framework concepts, variables, timing, and estimated burden

Concept	Variables and measures	Time points	Estimated burden
Sample characteristics	Age, sex/gender, race/ethnicity, marital status, education, relationship of FCG to care recipient, how long the FCG has been providing care to the recipient, any computer/smartphone experience and availability, diagnoses, medications	Baseline	2–6 min
Properties of the transition	Mutuality, single items for relationship of FCG/care recipient; caregiving demand	Baseline	2–6 min
Transition conditions (facilitators or barriers)	Spiritual/religious beliefs, time available for caregiving (FCG responsibilities and demands), decision-making preference, health literacy, income (single items), FCG support needs, hospital discharge readiness, FCG personal health problems	Baseline	5–12 min
Patterns of response (outcomes)			
Aim 1 (primary)	Preparedness for caregiving [36], communication skills, CAPACITY measure, satisfaction with care (PACIC)	Baseline, 2 weeks, and 8 weeks	6–10 min
Aim 2 (secondary)	CQOLC scale, coping, depression, burden (Bakas Caregiving Outcomes Scale), Palliative Care Outcomes Scale-Carer	Baseline, 2 weeks, and 8 weeks	17–20 min
Aim 3 (secondary)	Health care utilization and cost (Ambulatory and Home Care Record)	Monthly, 6 months	30 min

*Abbreviations*: *CAPACITY* Caregiver Perceptions About Communication With Clinical Team Members, *CQOLC* Caregiver Quality of Life-Cancer scale, *FCG* family caregiver, *PACIC* Patient Assessment of Chronic Illness Care

##### Part 2: At-home technology-enhanced intervention

The first video visit by the nurse and the FCG occurs within 24 to 48 h of hospital discharge. A minimum of 2 weekly video visits occurs with the FCG in the first 4 weeks after the care recipient’s discharge from the hospital. A minimum of 1 weekly visit occurs during the second 4 weeks after hospital discharge.

During visits, the nurse (1) provides ongoing education, guidance, and support to the FCG to develop caregiving and self-care and coping skills; (2) coordinates the implementation of the community-based aspects of the transitional care plan to address the FCG’s as well as the patient’s needs; (3) reaffirms and provides education and anticipatory guidance for the use of individualized plans for common care recipient symptoms, such as pain, breathlessness, and anxiety, as needed; (4) if requested, collaborates through active dialogue with the local community resources included in the transition plan; (5) if requested, collaborates with the primary care provider/specialty providers related to goals of care; (6) coaches the FCG in preparation for care recipient/provider office visits; and, based on continuing assessments; and (7) provides additional video visits/telephone calls to support the FCG, especially during care recipient health crises, impending death, death, or bereavement. Palliative and transitional care intervention objectives and activities are shown in Table 2. Frequency, type, and length of contacts are documented to quantify the dose effect of the intervention.

For video visits, Vidyo, a Health Insurance Portability and Accountability Act (HIPAA)-compliant software platform, is used with equipment provided by the study. The SC provides training to FCGs on how to use the tablet and software while the care recipient is still hospitalized.

The intervention continues with the FCGs during the 8-week period. On the basis of our prior experience with the FCG population, it is possible that the care recipient may be placed in a long-term nursing facility or die during the 8-week study period. In either case, the interventionist continues the intervention with the FCG. If the care recipient dies, bereavement support (telephone calls, video visits, and written educational materials) is offered for the duration of the 8-week intervention interval.

#### Control group: attention control condition

The control condition is based on usual care. Implementing a technology-enhanced model of care for transitional palliative care or attention control for adult FCGs in rural or medically underserved areas does not require alteration to usual care pathways (including use of medication). Usual care practice continues for both trial arms. Control group care recipients receive consult visits by the palliative care team while hospitalized, usual hospital discharge planning, and primary and specialty care in the community after hospital discharge. Data on services received by the FCGs are collected and considered for analysis as confounding factors. Care recipients do not participate in any study activities. To reduce attrition and optimize survey response rates, FCGs assigned to the control group receive monthly telephone calls from a team member to collect cost data. These calls serve as the attention control mechanism to minimize attrition and account for the nonspecific conditions of expectancy, social support, and attention considered necessary to generate placebo effects [37]. If concerns are identified during the attention control interaction between FCGs and study personnel, the FCGs are advised to call their primary care provider.

### Evaluation

#### Data collection and management

Multiple sources are used to collect data for the study. First, sociodemographic data and information on discharge disposition, diagnoses, and date of death from the care recipient’s medical record are recorded. Second, FCGs are asked to complete survey data at baseline (during the care recipient’s inpatient hospital stay) and then 2 weeks, 8 weeks, and 6 months after discharge. Baseline surveys are distributed during the hospital stay, but all subsequent surveys are mailed or emailed. If FCGs need or request additional help, the SC will assist by collecting data over the telephone. If the care recipient dies, we collect final data from the FCG approximately 2 weeks after the death, given that grieving family members’ distress may be more stable 2 weeks after their loved one’s death [38, 39]. Because of the risk of AEs associated with depression, any FCG returning a survey with a high score on the depression scale is contacted by telephone. For those in the intervention group, a nurse interventionist contacts the FCG. For those in the control group, the study’s social worker follows up. Third, an SC collects cost data from the FCG by telephone monthly for 6 months after each care recipient’s discharge from the index hospitalization to minimize recall bias and to account for any time lag in billing systems. Fourth, we use Nightingale Notes (Champ Software, Inc), a web-based and HIPAA-compliant clinical documentation system for the collection of the intervention activity. Nightingale Notes is based on the Omaha System, a research-based, comprehensive practice and documentation standardized taxonomy. Its structure enables relational data collection of assessments, interventions, and outcomes. By using this system, we maintain a record for the FCG that is independent of the care recipient’s electronic health record [40].

Throughout the trial, we evaluate procedures, such as checking the integrity of data storage and examining frequency distributions to look for anomalies such as an excessive number of “not applicable” or missing responses or problems with skip patterns. To ensure the reliability of the entered data, the PI reviews a random sample and compares the information recorded on the database program. An acceptable error rate is less than 0.3% (3 per 1000 entries). Quality assurance reports are prepared on an annual basis and reviewed by the PI and coinvestigators (Co-Is). The reports contain information about missing, invalid, or inconsistent data on selected key variables and participant dropout. The reports also contain a summary of key characteristics of the study participants and a summary of the completeness and quality of data.

#### Baseline and outcomes measures

Table 3 summarizes the framework concepts, variables and measures, and timing of data collection.

#### Demographics, transition properties, and conditions

Care recipient and FCG sociodemographic characteristics are abstracted from the electronic health record and the baseline survey. The transition properties include the quality of the FCG/care recipient relationship (Assessment of Mutuality [36, 41–44]), and FCG demands (hours of caregiving and length of time caregiving [45]) are used as covariates for analysis. Measures of facilitators/barriers for transition include spiritual/religious beliefs [46], time available for caregiving (due to other FCG responsibilities and demands) [45], decision-making preferences [47], health literacy [48], income, FCG support needs [49], readiness for hospital discharge [50] (measured at hospital discharge), and FCG personal health problems (if any) [45] and will also be used as covariates in modeling the intervention effects.

#### Primary outcomes

The study’s primary outcomes are associated with care quality (Table 3). Measures include preparedness for caregiving using the Preparedness for Caregiving Scale [36, 42, 43], FCG communication skills with providers using the Communication with Physicians scale [51] and Caregiver Perceptions About Communication With Clinical Team Members scale [52], and satisfaction with care, using a modified version of the Patient Assessment of Chronic Illness Care [53] to obtain FCG perceptions of the quality of chronic illness care received by the care recipient.

#### Secondary outcomes

Secondary outcomes include intrapersonal factors, such as FCG quality of life and well-being. Quality of life is measured with the Caregiver Quality of Life Scale–Cancer [54]. FCG burden is assessed using the Bakas Caregiving Outcomes Scale-Revised, which assesses changes as indicators of the effect of caregiving on FCGs’ lives [55]. Coping is measured with the Post-Discharge Coping Difficulty Scale to measure the degree of difficulty in coping with stress, recovery, self-care and management of medical needs, help and emotional support needed, confidence in self-care and medical management abilities, and adjustment after hospital discharge [56, 57]. Depression is measured with the Center for Epidemiologic Studies Depression Scale 10 [58–61].

Secondary outcomes also include time and monetary costs from a societal perspective, with costs from all stakeholders (FCG, care recipients, third-party insurers, and health systems) collected. Costs include self-reported utilization and cost information for care provided by FCGs and other unpaid caregivers, paid care received at home (e.g., home health care) and outside of the home (e.g., doctor/therapy appointments, emergency department visits and hospitalizations), and medications, supplies and equipment, and time costs. Time costs refer to the FCGs’ time and, if the FCG is employed, the employer time lost in the course of providing care to the care recipient. Time costs for the FCGs will be valued by a human capital approach [62]. The Ambulatory and Home Care Record (AHCR) [63] is used as our health care cost measure. The AHCR is designed to capture costs from a societal perspective, implying that costs from all stakeholders (FCG, care recipients, and health systems) are collected. Out-of-pocket costs refer to all care-related expenses not paid for by insurance (Table 4).

**Table 4:** Modified framework for assessment of palliative care costs, expenditure categories

Third party–incurred costs (commercial/Medicare/Medicaid/other/no insurance)	Privately incurred (out-of-pocket) costs	Time costs
Home-based services	Home-based services	Caregiver time losses from:
Ambulatory appointments	Ambulatory appointments	Labor market
Hospitalization	Hospitalization	Household work
Emergency department visits	Emergency department visits	Leisure
Facility care	Facility care	
Medications	Medications	Employer time loss
Supplies and equipment	Supplies and equipment	
	Paid housework	
	Travel expenses	
**Total third party expenditures**	**Total out-of-pocket expenditures**	**Total time cost**

Modified from Guerriere and Coyte [64]

For FCGs who are employed, the most recently available data on current earnings by age and sex from the US Bureau of Labor Statistics is used to impute the market value of time withdrawn from leisure and household work. To value lost time from the labor market, age- and sex-based earnings from the US Bureau of Labor Statistics are adjusted for employer-paid benefits, vacation days, and holidays. For FCGs who are not employed outside the home, their time lost from household work is imputed at the hourly earnings rate for the “personal care and service occupations” category in the US Bureau of Labor Statistics. Thus, the proposed valuation of the lost time is a function of whether the time is diverted from the labor market, household work, or leisure.

Because collection of cost data has many practical difficulties such as recall bias and difficulty understanding medical bills, we will also conduct a subgroup analysis of health care utilization (emergency department visits, inpatient hospitalization) and all-cause cost for FCGs who receive care within the primary care practice of the participating health system by using health care claims data from the practice. Patients enrolled in the health system are expected to receive all of their care within that system, and thus, the possibility of leakage in cost data due to receipt of care at outside providers is negligible. Cost data will be standardized per the Medicare reimbursement rate through a method outlined elsewhere [65].

#### Power and sample size calculation

Determination of sample size is based on the analyses of study aims 1 and 2 to detect a meaningful effect size. The focus of this study is FCGs. With a sample size of 100 FCGs per group, we have 80% power to detect an effect size of 0.487 at an alpha level of 0.01, assuming a 2-sided, 2-sample *t* test is appropriate. This suggests that, for a 1 SD of difference between 2 groups for a particular end point, a difference of 0.487 points per month between 2 groups for that end point is detectable with this sample size. Given that our primary outcome of interest is rate of change, only FCGs with at least 1 post-baseline measurement would be able to be evaluated. To account for a possible attrition rate of approximately 40% [66] due to unanticipated events such as loss to follow-up, we plan to recruit a total of 167 FCGs per group at an average of 8 FCGs per month over a planned 36-month enrollment period. nQuery Advisor Version 7.0 (Statistical Solutions Ltd) was used to calculate the sample size.

#### Analytical plan

Assessment of possible imbalances in the baseline covariates that may occur between the 2 groups, due to randomized intervention assignment, is made by comparing the baseline characteristics between the 2 groups. Per a modified intention-to-treat analysis, patients with at least 1 post-baseline measurement will serve as the principle analysis set for efficacy assessments.

##### Aims 1 and 2

Statistical analyses of aims 1 and 2 are performed similarly. Every FCG is evaluated using various scales and survey instruments at baseline, 2 weeks, and 8 weeks after patient discharge (Table 3). We use a response-feature analysis as our primary approach in analyzing these repeated measures data [67]. For each FCG, the magnitude of the scores from the respective instruments is plotted versus time in months; least-squares regression is used to estimate slope (i.e., a participant-specific rate of increase in points per month). This slope parameter estimate is used as the response feature for each participant and is the primary end point for this study. This primary end point is analyzed using a 2-sample *t* test or Wilcoxon rank sum test, as appropriate. The end points are also analyzed using a fixed-effects linear regression model to account for any baseline imbalances that are not accounted for by randomization. This model uses the estimated regression–slope response feature as the dependent variable and independent variables consisting of intervention (yes vs no) as well as any baseline covariates (e.g., FCGs’ age, employment status).

##### Aim 3

Descriptive statistics for the utilization measures (i.e., hospitalizations and emergency department visits) are provided. Considering the count nature of the utilizations, and to also account for any potential differences between the baseline characteristics of the care recipients in the 2 study arms, we use Poisson regression to model various utilization outcomes [68]. We expect that the gains (decreased health care utilization and costs) are sustained beyond the enrollment period. Therefore, both descriptive and multivariable-adjusted measures are estimated for the intervention period and the subsequent months post-intervention. A limitation of this economic approach is the memory recall of the FCGs. We provide a worksheet similar to Table 4 for all participants to help them track services and costs.

For analyses of costs, standard descriptive statistics including mean (SD) or median (range) are provided for all cost outcomes measured individually and also for the overall cost measure. The differences in overall cost between the intervention and control group provide an estimate of the cost impact of the transitional palliative care for FCG model of care. The difference in hospitalization and emergency department costs between the treatment and control arms determines the cost avoidance potential of implementing the intervention. The differences in FCG cost, a component of the overall costs, between intervention and control groups provide evidence of the impact of the intervention on this specific cost component. Both the descriptive and multivariable-adjusted measures are estimated for 8 weeks and 6 months. Because the distribution of health care costs and length of stay are generally skewed, both the length of stay and costs are log transformed before any estimation; the estimated coefficients can then be interpreted as the approximate percentage changes. Alternatively, we also explore modeling costs and length of stay with a generalized linear model with gamma distribution and log link [69]. Furthermore, to account for the possibility that some of the study patients die before the 6-month follow-up, we also apply econometric methods of censored regression [70, 71].

#### Data monitoring plan

A specific Data and Safety Monitoring Plan has been designed to effectively protect all data and any associated patient identifiers. The overall framework for safety monitoring includes procedures for monitoring study safety, minimizing research-associated risk, and protecting the confidentiality of participant data. A study manual containing standard operating procedures is used for training study staff and is available as a staff resource for the duration of the study. Compliance of regulatory documents and study data accuracy and completeness is maintained through an internal study team quality-assurance process. An intervention guide is developed to help ensure fidelity to the protocol. The PI and Co-Is meet with the Data and Safety Monitoring Committee before patient enrollment begins and semiannually thereafter, or more frequently if necessary or requested by the committee, at which time they present both a written and verbal report to the Data and Safety Monitoring Committee members. The report includes a summary of cumulative accrual and attrition, randomization, adherence to protocols, patient concerns, AEs, serious AEs, data completeness, and quality. Because of the processes used in the pilot study and lack of AEs due to study interventions and procedures, no interim analysis or formulation of stopping rules is planned.

#### Intervention fidelity

The investigators ensure that all study personnel are delivering the intervention according to the study procedure manual through weekly supervision/monitoring during the first month and monthly thereafter. An intervention guide is developed to help ensure fidelity to the protocol. To guarantee intervention fidelity, 2 of the Co-Is audit the first 6 intervention interactions and evaluate with a detailed checklist; 100% adherence is the expectation. Any inconsistencies or deviations from the protocol are addressed with the study team immediately. To monitor fidelity over the course of the study, Co-Is use a checklist to audit 2 intervention interactions for each FCG per study quarter. Challenges to intervention fidelity and how they are resolved are discussed with the intervention nurses during quarterly meetings. Review of study procedures and retraining of the intervention nurses and on-call palliative care staff are done by the investigators, as needed.

## Discussion

This study represents a substantial departure from current transitional and palliative care approaches that are limited to in-person interactions between a clinician and patient in both the hospital and home. Our care model advances an individualized approach to extending palliative care and provides transitional support for FCGs in distant, rural, underserved areas using HIT to continue face-to-face interactions. Our study not only addresses the critical and costly barrier imposed by distance, but also extends palliative care practice by improving transition management for the impending increase in the number of FCGs of seriously ill patients in rural areas through evolving demographic shifts [72].

This study proposes a novel HIT-enhanced intervention to support FCGs in the transition of their loved ones from hospital to home, which is based on FCGs’ unique needs rather than as an adjunct to patients’ needs. Recognition of FCG strengths in addition to needs is another unique feature of this study. Identifying individuals’ strengths is an important step in providing comprehensive, holistic care as a complement to a traditional problem-solving approach [73–76]. Moving from a needs-based to a whole-person perspective requires seeing individuals as whole persons with strengths that can be used to optimize well-being and caregiving [77].

This study fills another important and acute gap in the comprehensive assessment of the true financial burden borne by FCGs. This study helps define the value, types, and sources of resources used in providing palliative care, including the out-of-pocket costs for medications, supplies, care providers, travel expenses, and forgone time (time costs) for FCGs [63, 64, 78].

### Potential problems and alternative strategies

The primary challenges in completing this study in a timely manner are recruitment of FCG participants during their care recipients’ hospital stay and completion of data collection after hospital discharge. Previous studies have also described challenges in recruiting and retaining FCG study participants. We have identified several strategies to overcome these potential problems. One strategy is targeting recruitment during midday and early evening to facilitate patient and FCG availability. The SC first contacts FCGs via telephone to ascertain their interest in learning about the study and determine when the FCG will be available. The SC study telephone is exclusive to the study and is answered by the SC when questions arise. We also have strong, ongoing relationships with the Palliative Care consulting teams, who have ongoing relationships with the patients and their FCGs on their caseload. These clinically based partners encourage study participation.

To ensure maximal completeness of post-discharge data, the SC requests at least 2 telephone numbers (typically home and cell number) for all FCGs, as well as the name and telephone number of another person who may know their whereabouts. Follow-up calls to schedule data collection are made at the number and time of day the FCGs suggest. The SC-dedicated telephone also has caller identification recognizable to the participants to ensure completeness of follow-up data.

Data collection is completed on the basis of FCGs’ preferences using telephone interviewing or mailed questionnaire with a stamped, preaddressed return envelope or emailed survey. A cover letter accompanies the emailed and mailed questionnaire requesting that the FCGs complete the questionnaire on the same day they receive it, if possible. The SC’s telephone number is included, along with the offer to administer the questionnaire over the telephone if preferred. If at first we are unable to connect, telephone calls are made at varying times during the day. We also provide remuneration to all FCG participants commensurate with the time spent in the study (intervention activities for intervention group, data collection for both groups) to encourage continued engagement. If a care recipient dies during the data collection period, it may be difficult to collect subsequent data from the FCG. Collecting cost data by telephone instead of mail serves as an attention control device for FCGs in the study’s control condition group.

### Summary

Palliative care principles promote the unit of care to be the patient and family [79, 80]. The emerging literature, however, has recognized the profound physical, emotional, social, and financial impact of caring for a loved one with a life-limiting illness in the home environment, which has led to a call for increased support for the FCG transition experience [1]. Recognizing the critical contribution of and inherent challenges of caregiving for FCGs requires that their needs be addressed separately from those of the patients and be included in a plan of care [81]. Although the importance of FCGs for palliative care patients is well documented [82–85], interventions focused on FCGs in transitional care models are limited [24, 26, 86, 87]. Therefore, this study fills an important gap in the literature and will provide data on the financial impact of the intervention, which will allow for comparisons in costs that would be critical for further implementation.

## Trial status

Protocol version number 11. Recruitment began March 2017 and will be completed approximately fourth quarter 2020. Assessments continue until the end of 2021.

## Supplementary Information

**Additional file 1: Supplemental Table 1.** Technology-Enhanced Transitional Palliative Care for Family World Health Organization Trial Registration Data Set Information.

## Data Availability

The research will be disseminated at national scientific meetings, and manuscripts will be generated from the Specific Aims. Clean, deidentified datasets will be available to qualified investigators through communication of reasonable requests with the PI after primary results manuscripts are accepted.

## Abbreviations

AE: Adverse event
AHCR: Ambulatory and Home Care Record
APN: Advanced practice nurse
Co-I: Coinvestigator
DSMC: Data Safety Monitoring Committee
FCG: Family caregiver
HIPAA: Health Insurance Portability and Accountability Act
HIT: Health information technology
IRB: Institutional Review Board
PI: Principal investigator
SC: Study coordinator
