# Supplementary material for: A technology-enhanced model of care for transitional palliative care versus attention control for adult family caregivers in rural or medically underserved areas: study protocol for a randomized controlled trial
Source: Trials. 2020 Oct 28;21:895. doi: 10.1186/s13063-020-04806-0 (PMC7594268; doi:10.1186/s13063-020-04806-0)
Supplement: Supplementary file 1 — Additional file 1: Supplemental Table 1. Technology-Enhanced Transitional Palliative Care for Family World Health Organization Trial Registration Data Set Information. [file 13063_2020_4806_MOESM1_ESM.docx]

**Supplemental Table 1.** Technology-Enhanced Transitional Palliative Care for Family World Health Organization Trial Registration Data Set Information**.**

| **Data category** | **Information**[**^32^**](http://www.spirit-statement.org/spirit-statement/references#32) |
| --- | --- |
| Primary registry and trial identifying number | ClinicalTrials.gov NCT03339271 |
| Date of registration in primary registry | 13 November, 2017 |
| Secondary identifying numbers | R01NR016433; MC17-005188 |
| Source(s) of monetary or material support | NIH-NINR |
| Primary sponsor | Mayo Clinic |
| Secondary sponsor(s) | - |
| Contact for public queries | JC, CCRP [507-255-1036] [cogswell.jodie@mayo.edu] |
| Contact for scientific queries | JG, PhD Mayo Clinic |
| Public title | Technology-enhanced Transitional Palliative Care for Family Caregivers |
| Scientific title | Technology=enhanced Transitional Palliative Care for Family Caregivers in Rural Settings |
| Countries of recruitment | United States |
| Health condition(s) or problem(s) studied | Palliative Care |
| Intervention(s) | Intervention: Behavioral: Technology-enhanced Support  Comparator: Behavioral - Attention control |
| Key inclusion and exclusion criteria | Ages eligible for study: ≥21 years (Adult, Older Adult) Sexes eligible for study: both Accepts healthy volunteers: yes Inclusion criteria: adult family caregiver of adult patient hospitalized at Mayo Clinic Rochester; receives in-hospital palliative care consult; family caregiver lives in a Minnesota or Iowa county that is designated as medically underserved or rural area Exclusion criteria: Patient’s hospital discharge disposition include long-term placement (>2 weeks) inn a skilled nursing or hospice facility; patients with left ventricular assistive devices, documented chronic pain, use of home infusion pumps, or documented addictive behaviors. |
| Study type | Interventional Allocation: randomized Intervention model: parallel assignment Masking: double blind (subject, caregiver, investigator, outcomes assessor) Primary purpose: prevention Phase III |
| Date of first enrolment | March 2018 |
| Target sample size | 334 |
| Recruitment status | Recruiting |
| Primary outcome(s) | Change in Preparedness for Caregiving Scale Score  Change in Communication with Physicians Scale Score  Change in Caregiver Perceptions About Communication with Clinical Team Members Scale Score  Change in Patient Assessment of Chronic Illness Care (PACIC) Scale Score |
| Key secondary outcomes | Change in Caregiver Quality of Life Scale – Cancer (CQOL-C) Score  Change in Bakas Caregiving Outcomes Scale-Revised (BCOS-R) Score  Change in Post Discharge Coping Difficulty Scale (PDCDS) Score  Change in Center for Epidemiological Studies Depression Scale (CESD-10) Score  Mean Total Third Party Expenditures at 8 weeks  Mean Total Out-of-Pocket Expenditures at 8 weeks  Mean Total Time for Family Caregivers at 8 weeks |
